# Modelling Human Channelopathies Using Induced Pluripotent Stem Cells: A Comprehensive Review

**DOI:** 10.1155/2013/496501

**Published:** 2013-05-12

**Authors:** Martin Müller, Thomas Seufferlein, Anett Illing, Jörg Homann

**Affiliations:** ^1^Department of Internal Medicine I, University Hospital of Ulm, Albert-Einstein Allee 23, 89081 Ulm, Germany; ^2^Department of Internal Medicine II, University Hospital of Ulm, Albert-Einstein Allee 23, 89081 Ulm, Germany

## Abstract

The generation of induced pluripotent stem cells (iPS cells) has pioneered the field of regenerative medicine and developmental biology. They can be generated by overexpression of a defined set of transcription factors in somatic cells derived from easily accessible tissues such as skin or plucked hair or even human urine. In case of applying this tool to patients who are classified into a disease group, it enables the generation of a disease- and patient-specific research platform. iPS cells have proven a significant tool to elucidate pathophysiological mechanisms in various diseases such as diabetes, blood disorders, defined neurological disorders, and genetic liver disease. One of the first successfully modelled human diseases was long QT syndrome, an inherited cardiac channelopathy which causes potentially fatal cardiac arrhythmia. This review summarizes the efforts of reprogramming various types of long QT syndrome and discusses the potential underlying mechanisms and their application.

## 1. Introduction

“Inherited long QT syndrome” comprises a group of channelopathies that cause a delayed repolarization of the heart leading to an increased risk of malignant ventricular tachycardias, in particular *torsade de pointes,* that imply the risk of a fatal cardiac arrest. Several attempts have been made to estimate the prevalence of long QT syndromes in the past, while older studies quantify the prevalence between 1:20000 and 1:5000. The latest analysis by Schwartz et al. provides evidence for a higher prevalence close to 1:2000 in a Caucasian population [1]. It is assumed that up to 30% of sudden unexpected deaths in infants are caused by different forms of long QT syndromes (LQTS). These data also implicate that most cases of the LQTSs are diagnosed when they become clinically apparent in an individual or his/her family. Subclinical forms of LQT syndrome can become apparent under the influence of various drugs with QT elongation capability [2].

**Figure 1:**
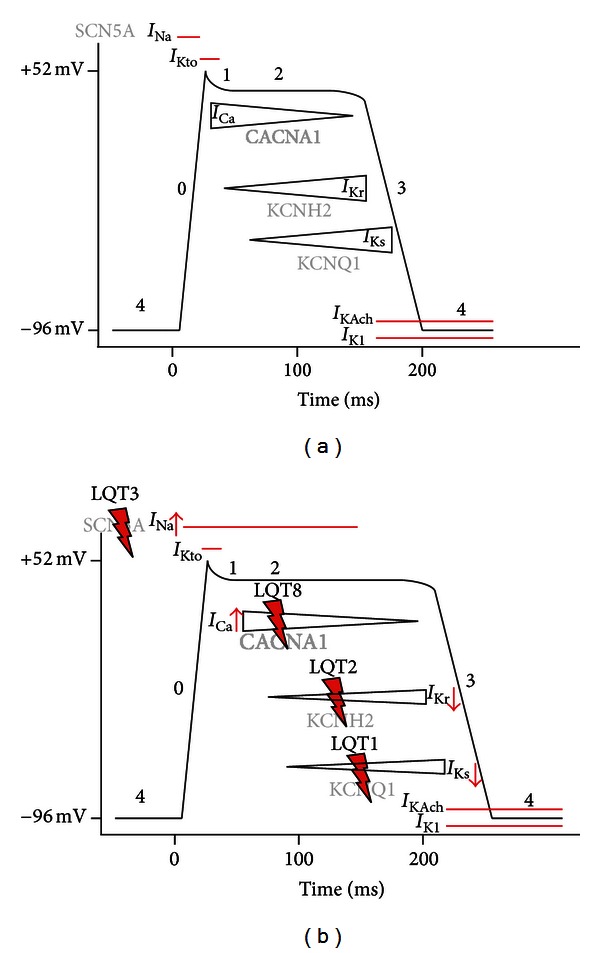
Schematic delineation of the cardiac action potential—resting “4,” upstroke “0,” early repolarization “1,” plateau “2,” and final repolarization “3.” Inward currents: *I*_Na_ and *I*_Ca_. Delayed rectifier currents: *I*_Kr_, *I*_Ks_. Inward rectifier currents: *I*_K1_, *I*_Kach_. Adapted from [3]: (a) normal cardiac action potential. Different currents are allocated to their chronology in the AP course. Ion channel genes are written semitransparent. (b) Different LQTSs are shown in relation to their distinct ion current causative for the indicated syndrome. Overactivation/reduction of different currents leads to a significant elongation of the action potential.

Ion channels represent a large group of pore proteins regulating ion efflux from the inner cell to the extracellular compartment or vice versa, thereby inducing changes in the membrane potential. Activity is mainly regulated either by voltage or by certain ligands. Thereby, a variety of ion currents are regulated in various tissues. Sodium, potassium, and calcium channels are the primary representatives of ion channel families in the human heart. A complex interplay of certain ion fluxes in a defined sequence operates the cardiac action potential. Thus, it is not surprising that slight mutations can disturb the ion pore structure, leading to changes in the currents' biophysical properties. Severe arrhythmia and eventually sudden cardiac death are the worst consequences. Those mutations causing human disease are so-called cardiac channelopathies. Different channelopathies are forming a basis of QT interval elongation, thereby increasing the susceptibility of electrophysiological deregulation of cardiomyocytes, particularly by decreasing the cardiomyocytes' ability of accurately timed repolarization. The myocardial action potential can be divided into 4 phases as follows (Figure 1): the primary depolarization in ventricular cardiomyocytes is characterized by a rapid increase of membrane conductance by Na^+^ channel (hNa_v_1.5 channel, encoded by the SCN5A gene) opening (phase1), followed by a light decrease of depolarization by subsequent opening of a special type of transient outward K^+^ channel (K_to_), which causes a short-lived, hyperpolarizing outward K^+^ current (*I*_Kto_). In a second phase, rapid repolarization is—in contrast to for example, neurons—impeded by a slow calcium influx (*I*_Ca,L_). Finally, repolarization is reached after closure of Ca^2+^ channels and when K^+^ (and therefore *I*_K_) increases, along with the inactivation of Ca^2+^ channels. Delayed rectifier K^+^ currents *I*_Kur_, *I*_Kr_, and *I*_Ks_ are slowly activating outward currents that play major roles in the control of repolarization. The deactivation of these channels is sufficiently slow so that they contribute outward current throughout phase 3 repolarization. Phase 4, or the resting potential, is stable at *≈*−96 mV in normal working myocardial cells and held up mainly by two inward rectifier channels *I*_KAch_ and *I*_K1_.

Mutations in long QT syndromes are consistently resulting in a relative increase of depolarizing currents against repolarizing ones (Figure 1). This results in two arrhythmia-promoting situations: (i) channels that remain depolarized for extended periods lead to increased refractory period, thus leading to areas of functional blocking which act as a reentry spot for ectopic excitation; (ii) as the elongation of action potential differs between epicardial (outer) and more endocardial (inner) cardiomyocytes, this may also promote the generation of functional reentry circles [2]. To date, 13 types of long QT syndromes are distinguished. Long QT syndromes are inherited either autosomal dominant or recessive with the recessive ones mostly having a more severe phenotype. Nonetheless, the penetrance in most long QT syndromes differs; as a consequence, there are individuals with mutations without any clinical appearance [4].

Upon expression of a defined set of transcription factors in somatic cells, iPS cells can be generated from virtually every type of tissue. The first human iPSCs were generated independently in 2007 by the Yamanaka [5] and the Thomson Laboratory [6]. Their unique features of unlimited self-renewal and nonrestricted differentiation power define a landmark in the context of understanding human development and disease [7–9]. More precisely, in case of applying this tool to patients who are classified into a disease group, it enables the generation of disease-specific iPS cells. iPS cells have proven a significant tool to elucidate pathophysiological mechanisms in various diseases such as diabetes, blood disorders, defined neurological disorders, and genetic liver disease [10–12]. iPS cells enable the dissection of monogenic human disease [13] mechanisms as well as mechanisms of genetically complex human disorders such as schizophrenia [14]. This opens promising perspectives both for the screening of innovative “druggable” targets [15] and *ex vivo* gene targeting therapies [13]. Moreover, a series of studies have successfully dissected a wide range of morphological and electric cardiac disease using patient-specific iPS cells as a model system [16–20]. In 2008, Mauritz et al. were the first to measure an AP from hiPSCs [21], followed by the first disease-specific study modelling LEOPARD syndrome [22]. Here, we summarize the current effort to model “electrical human cardiac disease” caused by channelopathies finally leading to LQT-syndromes: LQT1, LQT2, LQT3, and LQT8 (Table 1).

## 2. Modelling LQ Type 1 Syndrome

### 2.1. Pathophysiology

The highest incidence of all LQT syndromes is accredited to LQT1. It is characterized by clinical symptoms like adrenergic-induced *torsade de pointes* tachycardia, syncope, and effectiveness of *β*-adrenergic antagonistic “*β*-blocker” treatment. LQT1 accounts for about 50 percent of all genotyped patients with LQTs. Gene mutations in both KCNQ1 and KCNE1 lead to LQTS1. Thus, the myocardial sensitivity to catecholamine stimulation is increased by *I*_Ks_ reduction. As *I*_Kr_ (rapid component of the delayed rectifier current) can maintain normal duration of action potentials, LQT1 is often concealed. In those patients, the intake of *I*_Kr_ blocking drugs or hypokalaemia can lead to a burst of *torsade de pointes* by triggering a QT prolongation [4, 23]. *I*_Ks_ reduction leads to transmural dispersion of repolarization in the left ventricular wall; this dispersion may even be amplified by adrenergic stimulation as the resulting shortening of the action potential is emphasized in the epicardium and decreased in the midmyocardium [24]. This explains effectiveness of *β*-adrenergic blockade in LQT1.

### 2.2. The Model

Moretti et al. were the first group to publish a hiPSC model for LQTS. Moretti and her coworkers used fibroblast-derived iPSCs from two asymptomatic patients (father and son) with a KCNQ1-G569A mutation and cells from healthy controls. Differentiation of iPSCs was performed after embryoid body formation and consequent selection of areas of spontaneous contraction (indicative of cardiac differentiation). Finally, several different types of action potentials (atrial, nodal, and ventricular) were distinguished. Delayed rectifier currents were measured in specific ventricular-like cells: the cardiomyocytes (CM) derived from patient-specific LQT1 iPS cells showed reduced *I*_Ks_ peak and tail current densities, whereas *I*_Kr_ conductance appeared to be regular. APs of both atrial-like and ventricular-like hiPSC-CMs were significantly prolonged within in the LQT1 patient group compared to control cells. Only pacemaker-like cells showed no significant differences in AP periods. In 6 out of 9 LQT1-iPSC-CMs early afterdepolarisation (EAD)—as a proarrhythmogenic predicate—could be triggered by treatment with isoproterenol. No EADs could be triggered in WT-iPSC derived cardiomyocytes. The proarrhythmic effect of isoproterenol could be antagonized by admittance of *β*-blockers [25]. Thus, basic features of adult LQT-CMs could be reproduced.

## 3. Human iPS Cells Generated from Long QT Type 2 Syndrome

### 3.1. Pathophysiology

Next to LQTS1, LQTS2 represents the second most frequent genotype of LQTSs. About 40% of LQTS patients show aberrations in LQT2-associated gene locus for KCNH2 encoding the *α*-subunit of the *I*_Kr_ channel, linked to chromosome 7 [29]. Reduction of *I*_Kr_ slows and decelerates repolarization and, again, increases transmural dispersion by prolonging the action potential preferentially in the midmyocardium. A characteristic property of LQT2 is a faculty of arrhythmia induction by acute sympathetic activation like loud noise, anger, or other forms of emotional stress. These stimuli can acutely prolong the action potential and finally cause an enhancement of transmural repolarization heterogeneity. As bradycardia can also reduce *I*_Kr_ and, thus, lead to a delay of repolarization, arrhythmias can be triggered by both catecholaminergic-induced tachycardias and bradycardias. *β*-blockers can reduce the overall amount of cardiac events in LQT2 patients but they induce more cardiac events compared to the LQT1 collective [30–33]. On the other hand, LQT2 can be treated with controlled potassium supply as this leads to a reduction of QT dispersion and shortened QT intervals in those patients. 

### 3.2. The Model

First to report an iPSC-model of LQT2 were Itzhaki et al. in 2011 [15]. A patient- and disease-specific human iPSC line was generated from an individual with an A614 missense mutation in the KCN2 gene leading to LQT2, and cardiomyocytes were subsequently generated from those cells. Similar to the work previously published by Moretti et al. [25], three types of action potential morphologies were recorded from control- and LQTS iPSC-CMs: atrial-, nodal-, and ventricular-like, characterized by AP morphology. LQT2-derived cardiomyocytes showed marked prolongation of the action potential duration (APD). This prolongation persisted at different rates with external electrical stimulation. The LQT2 phenotype could even be recapitulated in control iPSC lines by pharmacologic inactivation of the *I*_Kr_ current with a specific blocker (E-4031). Single-cell voltage clamp studies identified the presence of an E4031-sensitive current (*I*_Kr_) in control human iPSC-CMs. Peak amplitudes of the *I*_Kr_ activation currents in LQTS cardiomyocytes were found to be significantly lower than in control cells. Even at multicellular level, the hiPSC-CMs produced a significant longer APD when compared to control cells. EADs could be found in both the atrial-like and ventricular-like LQT2 iPSC-CMs. APD prolongation could even be increased by E-4031 and cisapride in these cells. Moreover, Itzhaki et al. used their LQT2 iPSC-CMs as a platform for drug screens with a Ca^2+^ blocker (nifedipine), a K_ATP_-channel opener (pinacidil), and a Na^+−^ channel blocker (ranolazine). While nifedipine and pinacidil led to a significant shortening of the APD and could completely abolish triggered arrhythmias in LQT2 iPSC-CMs on multicell level, ranolazine reduced triggered arrhythmias but had no influence on APD, probably because of its nonspecific blocking effect on various ion channels [15]. Matsa et al. generated hiPSC-CMs from both symptomatic and asymptomatic patients with a G1681 mutation in KCNH2 and put more emphasis on iPS-based disease modelling as a drug screen platform. The LQT patients in this work (mother: asymptomatic, QTc interval 445 ms; daughter: symptomatic, QTc interval 571 ms) showed contrarious phenotypes despite the same mutation. In the symptomatic patient, syncope-like events took place after arousal from sleep, as typical for LQT2. hiPSC-CMs originated from skin fibroblasts. Interestingly, APDs from the mother's hiPSC-CMs were shorter than the daughter's thus reflecting the *in vivo* penetrance. Application of a sympathetic stimulus with isoprenaline led to electrophysiological abnormalities, for example, EADs, in 25% of the LQT2-hiPSC-CMs. These arrhythmias could be antagonized by *β*-blockers [26]. Lahti et al. used skin biopsy-derived iPSCs of an asymptomatic individual with a missense mutation in KCNH2 causing arginine-to-tryptophan substitution at position 176 (R176, hERG-FinB). CM differentiation was performed with WT-hiPSC-CMs, LQT2-hiPSC-CMs, and hES-CMs. Again, APs were divided into “atrial-like” and “ventricular-like.” Only ventricular-like APs showed a significant elongation, especially at low frequencies. EADs were observed in 1 out of 20 LQT2-hiPSC-CMs and were never observed in WT- hiPSC-CMs. Blocking of *I*_Kr_ channels with E4031 lead to an increase in EADs in both LQT2 and wild-type-derived cell lines, but this effect turned out to be more emphasized in LQT2-hiPSC-CMs [27].

## 4. A Long QT Type 3 Syndrome Model

### 4.1. Pathophysiology

LQT3 has a vastly lower incidence than LQT1/2, it is evident in about 7-8% of genotyped patients with LQTS [34]. Patients with LQT3 suffer from fatal cardiac events typically at night without excitation or arousal. Interestingly, only infrequently preliminary sympathetic stimulation can be found before cardiac events [35]. Surface ECG in LQT3 shows a flat, long ST segment with a late appearance of a narrow-peaked T wave [4, 36]. SCN5A mutations lead to gain of function of Na^+^ channel activity. Briefly, late sustained Na^+^ currents, slowed rate of inactivation, faster recovery from inactivation, and abnormal interaction with the channel's *β*-subunit define the mode of action in LQT3 syndrome [36]. Thereby, the plateau phase of the action potential is prolonged, producing long ST segments and later appearance of T wave in the ECG. Compared to patients with LQT1/2, *β*-blocker treatment is less effective in LQT3, for example, as that might prolong action potentials due to bradycardia [37]. On the other hand, Na^+^ channel blockers like mexiletine and flecainide can shorten the action potential both *in vitro* and *in vivo *[38]. Mexiletine leads to a shortened QT interval, a normalization of the T wave, and can even prevent *torsade de pointes* in short-time course. Atrioventricular block can also be improved. The effect of flecainide seems to be more mutation specific and it can induce Brugada type ECG changes. Once diagnosed, patients require implantation of a cardioverter defibrillator (ICD) because of the high incidence of malignant arrhythmias. Moreover, the pacemaker-function of the ICD helps to prevent arrhythmia-inducing bradycardias. 

### 4.2. The Models

Generation of LQT3-miPSC-CMs was first performed in 2011 by Malan et al. [28], this work is based on murine cells with deletion of the amino acids lysine-proline-glutamine in the intracellular loop between domains III and IV of the cardiac Na^+^ channel (SCN5). Patch clamp measurements of LQT3-miPSC-CMs showed faster recovery from inactivation and larger late currents than observed in controls. Duration of AP was prolonged; also EADs could be provoked at low pacing rates. 

Davis et al. generated iPSC lines from mice carrying the Scn5a (1798insD/+) (SCN5a-het) mutation [17]. In humans, the underlying mutation causes an overlap syndrome with clinical features of both LQT3 and Brugada syndrome. This work addresses the question whether relatively immature iPSCs-derived CMs can truly model gain- and loss-of- function genetic disorder affecting the Na^+^-current (*I*_Na_) in the face of their immaturity. Patch-clamp experiments showed that the SCN5-het cardiomyocytes had a significant decrease in *I*_Na_ density and a larger persistent *I*_Na_ compared with SCN5a-WT cells. AP measurements indicated longer APD in SCN5-het-derived CMs. Interestingly, these characteristics recapitulated the findings of isolated cardiomyocytes from adult mice. Patch-clamp measurements on the derivative cardiomyocytes revealed changes similar to those in the mouse iPSC-derived cardiomyocytes [17].

## 5. LQT8 (Timothy Syndrome)

LQT8, also named Timothy syndrome, was first described by Marks et al. and Reichenbach et al. [39, 40]. Compared to the named above types of LQTS, this syndrome manifests with major phenotypic abnormalities in multiple-organ systems, including skin, eyes, teeth, immune system, and brain. The majority of affected children die at an average age of 2.5 years. All affected individuals have severe prolongation of QT intervals, syndactyly, and abnormal teeth. Cardiac arrhythmias are the most serious aspect of this disorder: patients show QT prolongation, 2:1 atrioventricular block, T wave alteration, and life-threatening polymorphic ventricular tachycardias. In 2004, Splawski et al. [41] could specify the phenotypic characterization of the Timothy syndrome and finally attribute its variegated clinical attributes to a *de novo* missense mutation in the Ca_V_1.2 L-type calcium channel gene: Analysis of the affected patients Ca_V_1.2 splice variant revealed a G121A transition in exon 8A. The Ca_V_1.2 gene is expressed in multiple tissues. The disease-associated mutation causes abnormal Ca^2+^ currents. The mutated Ca^2+^ channel loses its voltage-dependent inactivation leading to sustained *I*_Ca,L_ action potential prolongation and Ca^2+^ overload. The consequence is a spontaneous Ca^2+^ release from the sarcoplasmatic reticulum and thus is a promotion of early delayed afterdepolarizations. It has been shown in single cases that treatment with Ca^2+^-channel blockers like verapamil can reduce the risk of arrhythmias. 

### 5.1. The Model

In 2011, Yazawa et al. have generated hiPSC-CMs from two Patients suffering from LQT8/Timothy syndrome due to an amino acid substitution in exon 8a of *CACNA1C*, the gene encoding Ca_V_1.2. The APs from iPSC-derived ventricular cells were three times longer than in controls, and EBs from those cells contracted only at 30 bpm (controls 60 bpm). Electrophysiological recordings and Ca^2+^ imaging studies of these ventricular-like cells showed irregular contraction, excessive Ca^2+^ influx as a source of prolonged action potentials, irregular electrical activity, and abnormal calcium: LQT8-iPSC-CMs showed a delay in inactivation of *I*_Ca,L_. The great impact of this work is outlined by the rescue experiments. Briefly, roscovitine, a compound that increases the voltage-dependent inactivation of Ca_V_1.2, restored the electrical and Ca^2+^ signalling properties of cardiomyocytes from Timothy syndrome patients [20, 42].

## 6. Conclusion

Several limitations of iPS-derived CMs have to be overcome. To date, most models mentioned previously lead to an “immature” electrophysiological phenotype, reminding more of fetal than adult CMs. As in most iPS cell-based disease models, further limitations come due to deficient purity of the cell populations. To date, these are not exceeding 50% purity of CMs. Beyond that, iPC-derived “CMs” comprise mixed cardiac subpopulations with various AP characteristics. 

**Figure 2:**
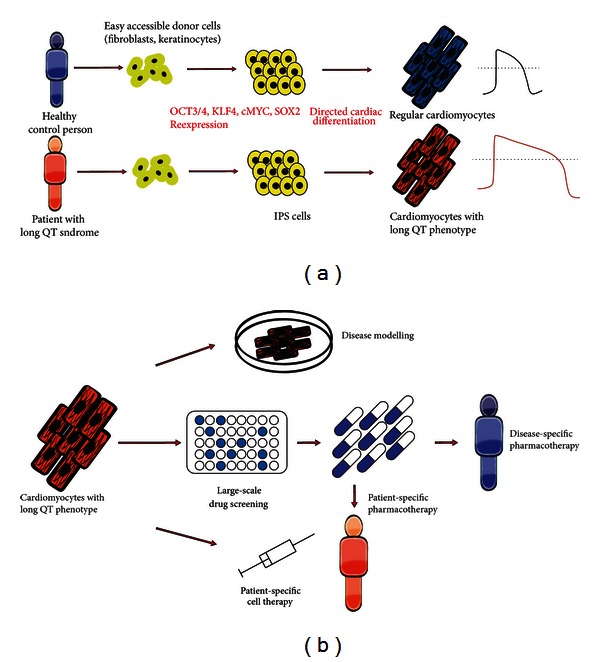
(a) Schematic presentation of iPSC-derived cardiomyocytes' retrieval. Based on easy accessible donor cells, like, for example, fibroblasts and consequent overexpression of different pluripotency factors, reprogrammed donor cells fall into a state of pluripotency (iPS-Cells). By various forms of directed cardiac differentiation, cardiomyocyte-like cells show essential characteristics of adult cardiomyocytes, maintaining their LQT-/non-LQT-phenotype. (b) Possible applications for iPSC-derived cardiomyocytes: (i) disease modelling for better understanding of genetic and epigenetic causation of LQTS; (ii) large-scale drug screening for both patient-specific and nonspecific pharmacotherapy; (iii) circumventing transplantation-associated immunogenicity by patient-specific cell therapy.

Nevertheless, human disease-specific iPS cells can be used to model different types of long QT syndrome. Not only the generated cardiomyocytes recapitulated human disease phenotype but also allowed the development of potential rescue strategies. In fact, this points to major application of human disease-specific iPS cells, namely, the opportunity of drug development in a disease-specific setting. Thereby, a variety of human diseases have been successfully studied while the vast majority of rescuing strategies were based on an educated guess. In the future, large-scale screening approaches using small molecule, shRNA, or cDNA libraries will shed a deep light on the pathophysiology of human disease and allow the development of specific drugs (Figure 2). 

## Figures and Tables

**Table 1:** Current iPS cell-based models for long QT syndromes.

LQTS subtype	Gene mutation	Protein	iPSC-C model
LQTS1	KCNQ1	Alpha-subunit of the delayed rectifier (slow) potassium channel (*I*_ks_)	Moretti et al., 2010 [25]
LQTS2	HERG	Alpha-subunit of the delayed rectifier (rapid) potassium channel (*I*_kr_)	Itzhaki et al., 2011 [15], Matsa et al., 2011 [26], Lahti et al., 2012 [27]
LQTS3	SNC5A	Alpha-subunit of the cardiac sodium channel	Malan et al., 2011 [28], Davis et al., 2012 [17]
LQTS8	CACNA1c	Alpha-1c-subunit of the L-type calcium channel	Yazawa et al., 2011 [20]

## Abbreviations

AP: Action potential
APD: Action potential duration
CM: Cardiomyocyte
EB: Embryoid body
EAD: Early afterdepolarization
ECG: Electrocardiogram
hESC: Human embryonic stem cell
hiPSC: Human-induced pluripotent stem cell
ICD: Implantable cardioverter defibrillator
miPSC: Murine-induced pluripotent stem cells
LQT(S): Long QT (syndrome)
QT-Interval: Time between start and end of cardiac ventricular electrical depolarization.
